# The Effects of Aquaporin-1 in Pulmonary Edema Induced by Fat Embolism Syndrome

**DOI:** 10.3390/ijms17071183

**Published:** 2016-07-21

**Authors:** Yiwei Zhang, Kun Tian, Yan Wang, Rong Zhang, Jiawei Shang, Wei Jiang, Aizhong Wang

**Affiliations:** Department of Anesthesiology, the Sixth People’s Hospital, Shanghai Jiao Tong University, Shanghai 200233, China; 1030218497@sjtu.edu.cn (Y.Z.); tiankun5282@126.com (K.T.); wangyanxq1989@163.com (Y.W.); lotuszhangrong@163.com (R.Z.); shangjiawei@sina.com (J.S.); jiangw@sjtu.edu.cn (W.J.)

**Keywords:** AQP1, fat embolism syndrome, pulmonary edema, free fatty acid, p38 MAPK signaling pathway

## Abstract

This study was designed to investigate the role of aquaporin1 (AQP1) in the pathologic process of pulmonary edema induced by fat embolism syndrome (FES) and the effects of a free fatty acid (FFA) mixture on AQP1 expression in pulmonary microvascular endothelial cells (PMVECs). In vivo, edema was more serious in FES mice compared with the control group. The expression of AQP1 and the wet-to-dry lung weight ratio (*W*/*D*) in the FES group were significantly increased compared with the control group. At the same time, inhibition of AQP1 decreased the pathological damage resulting from pulmonary edema. Then we performed a study in vitro to investigate whether AQP1 was induced by FFA release in FES. The mRNA and protein level of AQP1 were increased by FFAs in a dose- and time-dependent manner in PMVECs. In addition, the up-regulation of AQP1 was blocked by the inhibitor of p38 kinase, implicating the p38 MAPK pathway as involved in the FFA-induced AQP1 up-regulation in PMVECs. Our results demonstrate that AQP1 may play important roles in pulmonary edema induced by FES and can be regarded as a new therapy target for treatment of pulmonary edema induced by FES.

## 1. Introduction

Fat particles released into circulation after bone fracture or other trauma can cause a series of pathophysiological changes. These changes result in a clinical syndrome called fat embolism syndrome (FES). The main symptoms include dyspnea and hypoxemia [1]. Due to the physiological characteristics of the circulatory system, the fat particles reach the pulmonary vessels first and block the pulmonary capillaries and small blood vessels, leading to lung tissue hypoxia, capillary permeability increase and pulmonary edema. At the same time, the fat particles are decomposed into free fatty acids (FFAs), which can induce lung damage and cause dyspnea in FES patients in the early period [2].

However, the exact mechanism responsible for the development of pulmonary edema induced by FES remains unclear. Recent evidence suggests that aquaporins (AQPs) are expressed in the lung and may play a critical role in water transport [3,4]. Aquaporin1 (AQP1) is expressed in the capillary endothelium. It is important for the liquid exchange between the alveoli and capillaries [5]. The expression of AQP1 in the lung is decreased in a viral infection model of pulmonary edema [6,7]. As a result, we hypothesized that AQP1 might be involved in pulmonary edema induced by FES. In this study, we investigated the association of AQP1 expression with the pathological features of pulmonary edema in FES mouse models and then demonstrated the potential mechanism of FFA-induced AQP1 regulation in pulmonary microvascular endothelial cells (PMVECs).

## 2. Results

### 2.1. Fat Embolism Syndrome (FES) Model Was Established in the Mice

**Figure 1:**
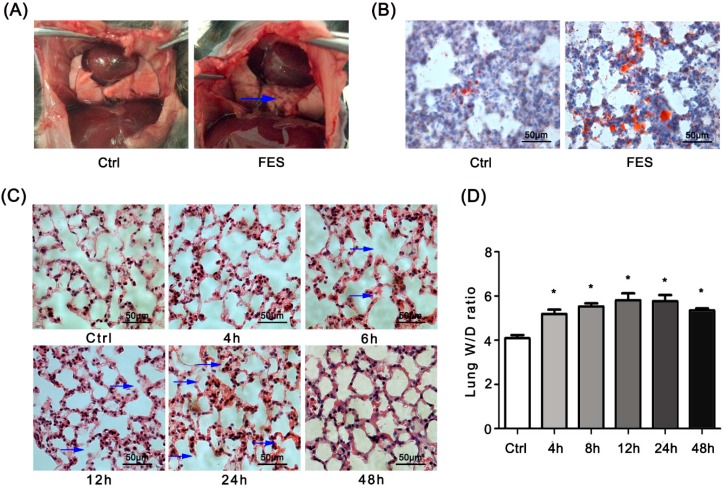
Pulmonary edema occurs in FES mice. (**A**) Gross morphology of the lungs in the control and FES (fat embolism syndrome) groups. Blue arrow, infarction tissues; (**B**) Lung sections from the control and FES groups were stained with Oil Red O; (**C**) Lung sections from the FES group at different time points after fat injection were stained with H & E. Blue arrow, ruptured alveolar wall; (**D**) *W*/*D* ratio in the FES group at different time points after fat injection. * *p* < 0.05, the statistics were made by comparing with Ctrl group, respectively.

The gross morphology of the FES groups showed an obvious scattered dark red infarction (Figure 1A). In the lung specimens, there were red free fat substances on Oil Red O staining in the blood vessels of the lung. In addition, the free fat substances had infiltrated the vascular wall (Figure 1B). While the microscopic morphology of the control group showed a clear and complete structure, the FES group showed great pulmonary edema with a widened interstitial space, ruptured alveolar wall and alveolar infiltration of inflammatory cells (Figure 1C). As early as 4 h, the lung *W*/*D* ratio in the FES group was already much higher than that in the control group (Figure 1D). These data showed that the FES model was successfully established.

### 2.2. AQP1 Is Increased in FES Mice

**Figure 2:**
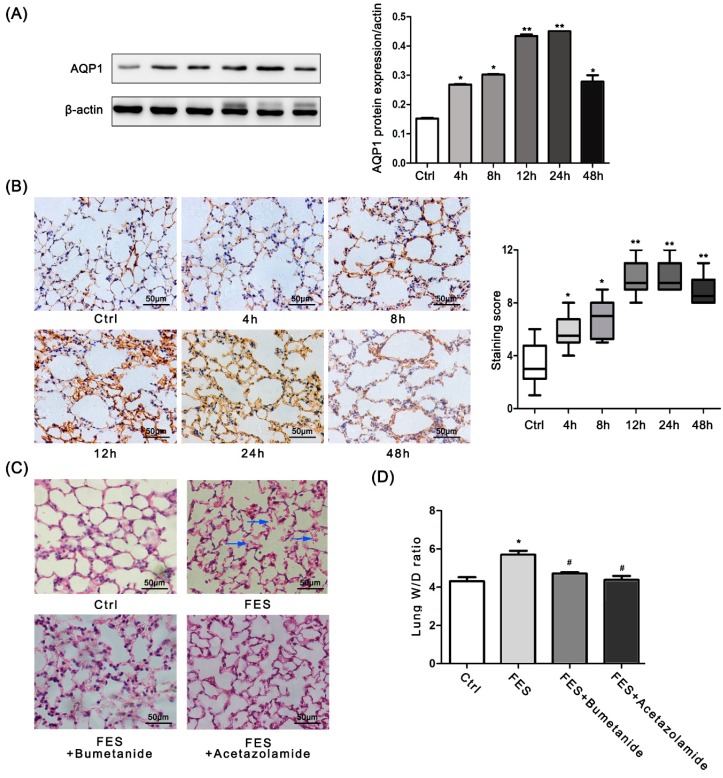
AQP1 is increased in lung of FES mice and inhibition of AQP1 reverses pulmonary edema in FES mice. (**A**) Western blot and (**B**) immunohistochemical analyses of AQP1 expression in the FES group at different time points after fat injection. Staining score was shown on the right; (**C**) Lung sections from the control, FES and FES + AQP1 inhibitors (bumetanideand acetazolamide, respectively) groups were stained with H&E. Blue arrow, ruptured alveolar wall, infiltration of red blood cells, and widened alveolar septa; (**D**) *W*/*D* ratio of the control, FES, FES + bumetanide and FES + acetazolamide groups. * *p* < 0.05; ** *p* < 0.001, the statistics were made by comparing with Ctrl group, respectively. ^#^ *p* < 0.05, the statistic was made by comparing with FES group.

AQP1 is located in the capillary endothelium and plays an important role in the liquid exchange between the alveoli and capillaries. To understand whether AQP1 was involved in the FES, we investigated the protein expression of AQP1 in the lungs of the FES mice. Western blot analysis revealed that AQP1 was significantly elevated in the FES group compared to the control group (Figure 2A). The immunohistochemical (IHC) assay also confirmed that AQP1 was up-regulated in the lungs of the FES mice (Figure 2B), which was consistent with the data from the Western blot. These data suggest that AQP1 expression was increased in FES.

### 2.3. AQP1 Is Required for the Lung Injury Induced by FES

In the control group, the alveolar septa were orderly and the cell morphology was normal, while in the FES group, the alveolar septa were widened without continuity, and a severe infiltration of red blood cells was observed. However, the AQP1 inhibitor significantly recovered the lung tissue morphology (Figure 2C). Moreover, the lungs in the FES group had a significantly increased *W*/*D* ratio at 24 h, and the ratio was reversed by pretreatment with AQP1 inhibitors (Figure 2D).

### 2.4. Morphological Characterization of Rat Pulmonary Microvascular Endothelial Cells

**Figure 3:**
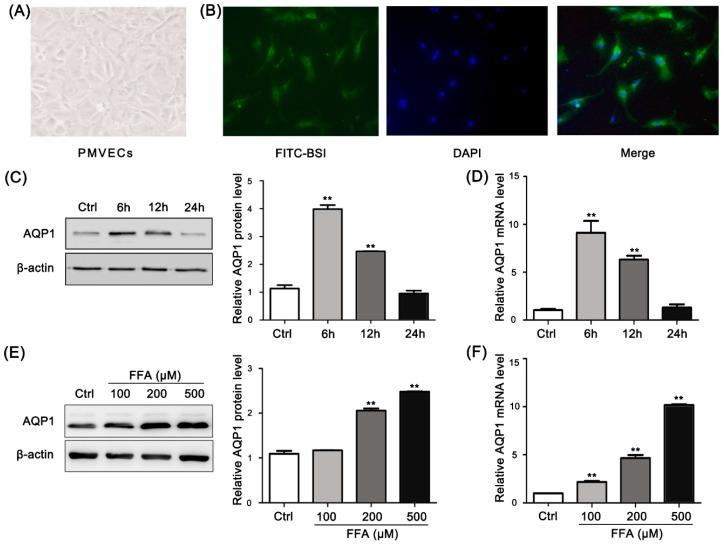
FFA induced up-regulation of AQP1 expression in PMVECs. (**A**) Primary cultured PMVECs obtained from normal rats. The PMVECs were polygonal or fusiform with a uniform size and displayed a similar and typical cobblestone-like morphology. Magnification 200×; (**B**) Fluorescence microscopy showed that the PMVECs exhibited green fluorescence after staining with FITC-BSI. The nuclei were stained blue by DAPI. Magnification 200×; (**C**,**D**) The protein and mRNA levels of AQP1 in PMVECs stimulated by 500 μM FFAs for different times; (**E**,**F**) The protein and mRNA levels of AQP1 in PMVECs stimulated by different concentrations of FFAs for 6 h. ** *p* < 0.01, the statistics were made by comparing with Ctrl group, respectively.

The cultured cells obtained from rats exhibited polygonal or fusiform morphologies under the inverted microscope. The cells displayed typical cobblestone-like morphology after their fusion to a confluent monolayer (Figure 3A). A recent study demonstrated that *Bandeiraea* *simplicifolia* isolectin (BSI) selectively interacted with PMVECs, particularly in vivo and in vitro [8]. The FITC-BSI assay revealed the positive findings (Figure 3B) under fluorescence microscopes.

### 2.5. Free Fatty Acid (FFA) Induces Up-Regulation of AQP1 in PMVECs

FFAs increased AQP1 expression in PMVECs in a time- and dose-related manner. To determine the AQP1 changes caused by FFAs at different time points, the cells were exposed to 500 μM FFAs for 6, 12, or 24 h. After 6 and 12 h, AQP1 protein was significantly (*p* < 0.05) increased compared with the control group (Figure 3C,D). The cells were treated with 0, 100, 200, and 500 μM FFAs for 6 h. The concentrations of 200 and 500 μM FFAs significantly (*p* < 0.05) increased the mRNA and protein levels of AQP1 compared with the control group (Figure 3E,F). AQP1 mRNA expression was maximally increased by 500 μM FFAs.

### 2.6. ERK, p38 Kinase, and JNK Activation by FFAs in PMVECs

**Figure 4:**
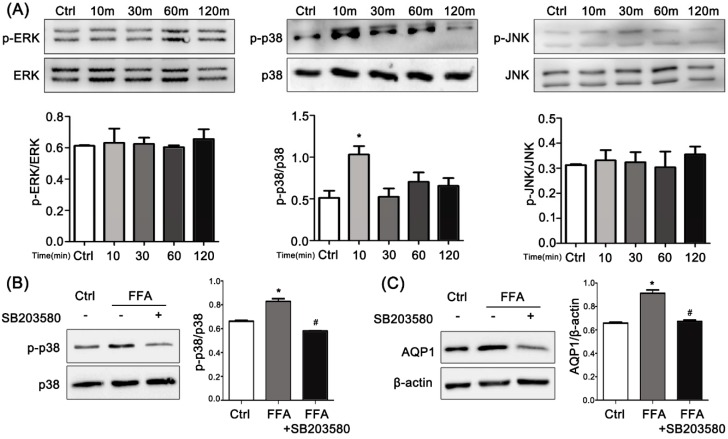
Activation of phosphorylation of MAPK by FFAs and effects of p38 inhibition on FFA-induced up-regulation of AQP1 in PMVECs. (**A**) Western blot analysis of p-ERK, p-p38 kinase, p-JNK expression in PMVECs stimulated by FFAs. P-p38 kinase was significantly activated; (**B**) SB203580 was confirmed to inhibit the expression of p-p38 kinase in PMVECs stimulated by FFAs; (**C**) Expression of AQP1 was reduced by SB203080 in PMVECs stimulated by FFAs. * *p* < 0.05, the statistics were made by comparing with Ctrl group, respectively. ^#^ *p* < 0.05, the statistics were made by comparing with FES group.

Our next objective was to define the potential signaling pathways by which FFAs up-regulated AQP1 expression in PMVECs. To determine whether MAPK-mediated signaling was involved in the up-regulation of AQP1 by FFAs, antibodies for the phosphorylated or the total form of the three MAPKs (p38/ERK/JNK) were used in experiments. PMVECs were treated with 500 μmol/L FFAs for 10, 30, 60 or 120 min. Then the phosphorylation of the three MAPKs was investigated by immunoblot analysis (Figure 4A). Low basal levels of phosphorylation of the MAPKs were detected in unstimulated PMVECs. Upon FFA stimulation, only p-p38 was markedly elevated, and p-JNK and p-ERK were not affected. These results strongly suggest that FFA significantly activated the phosphorylation of p38 in PMVECs.

### 2.7. Effects of p38 Inhibitor on FFA-Induced Up-Regulation of AQP1 in PMVECs

To further explore whether the activation of p38 MAPK pathways was involved in FFA-induced up-regulation of AQP1 in PMVECs, SB203580, an inhibitor of p38 kinase, was used. The PMVECs were first pretreated with 10 μM SB203580 for 30 min. FFA-induced p-p38 kinase was analyzed by Western blot analysis. As shown in Figure 4B, the p38 kinase inhibitor reduced the amount of p-p38 and effectively blocked the activation of the p38 pathway. Based on the results in Figure 4B, we proceeded to investigate the effects of the p38 kinase inhibitor on FFA-induced AQP1 protein expression and assessed whether activation of p38 kinase was actually involved in mediating the up-regulation of AQP1 by FFAs. The cells were pretreated with or without p38 kinase inhibitor and then stimulated with FFAs. In the presence of SB203580, AQP1 protein was significantly decreased compared with FFAs alone (Figure 4B). These data suggest that p38 kinase inhibitor strongly suppressed the FFA-induced up-regulation of AQP1 in PMVECs. These results clearly demonstrate that the p38 pathway is involved in the FFA-induced up-regulation of AQP1 expression in PMVECs.

## 3. Discussion

Fat particles enter the circulation and lead to a series of signs and symptoms of a clinical syndrome, which is called FES. FES often occurs after long bone or pelvic fractures. It can also be secondary to body and other fatty tissue trauma, such as liposuction [9]. Fat particles gathered in pulmonary capillaries and small blood vessels lead to pulmonary hypertension. At the same time, under the activation of fat enzymes induced by stress, fatty acids decompose and release a high concentration of FFAs, which results in platelet aggregation, slight DIC (disseminated intravascular coagulation) and pulmonary capillary burst. Lung histology demonstrates alveolar hemorrhage, damage to lung alveolar epithelial cells and pulmonary edema [10].

To the best of our knowledge, this is the first study to show the effects of AQP1 in FES. As we know, AQPs are a family of water-selective channels. They function to increase plasma membrane water permeability and facilitate water transmembrane transport [11]. AQP1 is one subtype of AQPs that is located in microvascular endothelial cells. AQP1 and AQP5 are the main water channel proteins in the lung. They provide a major pathway for driving water movement osmotically across epithelial and microvascular barriers in the lung [12]. A previous study showed fluid movement was reduced 10-fold by the deletion of AQP1 or AQP5 alone and reduced even more by the deletion of AQP1 and AQP5 together [13]. A change in either AQP1 or AQP5 expression may represent a response to inflammation-associated pulmonary edema, and it may be causal in the formation of pulmonary edema [14,15].

Our research used allograft perinephric fat of mice to make a FES model closely related to clinical FES. The changes in lung tissue morphology showed that alveolar septum fracture and the lung tissue structure were damaged 4 h after the injection of fat. The alveolar interval was broken and thickened. We also found red blood cells in the alveoli, especially at 12 h and 24 h. Furthermore, the trend of the *W*/*D* ratio demonstrated that pulmonary edema appeared 4 h after fat injection and continued to 48 h. Though the *W*/*D* ratio at 48 h was less than that at 24 h, it was still higher than that of the control group. After 48 h, the pulmonary edema subsided but never completely faded. Our lung tissue pathology morphology results are different from the conclusion McIff et al. [16] drew from a rat model. The differences may be the result of the type of animal and the dose of fat.

Our study showed a significantly increased expression of AQP1 in the mouse lungs in the FES model, which was accompanied by an increased *W*/*D* ratio. The immunohistochemical results showed that AQP1 was strongly expressed in the pulmonary microvascular endothelial cells, in line with the literature [17,18]. Water permeability between endothelial cells and alveoli is decreased by 90% in the lungs of AQP1-knockout mice. Knocking out AQP1 can decrease fluid accumulation in the lung due to hydrostatic pressure [19,20]. This may be the result of the reduction of AQP1 in the microvascular endothelial cells, which prevents water from entering the alveolar interval from vessels. To test this hypothesis, we chose the time at which AQP1 was obviously increased to perform further tests. Twenty-four hours after the injection of the AQP1 inhibitor, the *W*/*D* ratio and lung tissue pathology showed that the edema and structure of the lung had recovered to a certain degree. This indicates that increased AQP1 could promote edema and that AQP1 may be involved in the recovery process of FES lung injury.

To investigate the mechanism of the FES-induced alteration in AQP1 expression, we chose FFAs for our in vitro study. FFAs up-regulated AQP1 in PMVECs in a dose- and time-related manner. The increase in AQP1 mRNA was accompanied by a similar increase in protein expression. The liquid exchange between the alveoli and capillaries is influenced by the alterations of the expression of AQP1 [21,22]. The mechanism of edema in cells or tissues may be due in part to this relationship. The regulation of AQP1 expression in PMVECs has not been studied, though AQP1 can be regulated by MAPKs in various cells [23,24,25]. Here we detected the ERK/JNK/p38 pathways and found that only p38 was activated by FFAs via increased phosphorylation constituents. The FFA-induced up-regulation of AQP1 was suppressed by SB203580. These results imply the p38 signaling pathway is involved in the up-regulation of AQP1 by FFAs in PMVECs. Our results demonstrate that when FFA concentrations increase in pulmonary microvessels, it can phosphorylate p38 in PMVECs, resulting in AQP1 up-regulation. By integrating the in vitro and in vivo studies, we have revealed the mechanism of increased AQP1 expression in FES.

There are no previous reports describing the effects of AQP1 in pulmonary edema induced by fat embolism syndrome. Here we have provided the first evidence that AQP1 expression is increased in FES and that FFAs induce the up-regulation of AQP1. The findings from the current study indicate that the altered expression of AQP1 in FES may be associated with the development of pulmonary edema. To our knowledge, fluid accumulation around the airways could result either from increased extravasation of fluid across the endothelium, or from decreased clearance of fluid from the interstitial space. Consistent with our findings, a previous study found that decreased pulmonary vascular permeability was in AQP-1-null humans, which demonstrated increased AQP1-mediated fluid extravasation from vessel to interstitium [26].

## 4. Materials and Methods

### 4.1. Ethics Statement

This study was approved by the ethical committees of the 6th People’s Hospital affiliated Shanghai Jiaotong University (SYXK (Shanghai, China) 2011-0128, 1 January 2011). All animal experiments were conducted under the guidelines on humane use and care of laboratory animals for biomedical research published by National Institutes of Health (No. 85-23, revised 1996, 1 January 1996).

### 4.2. Mouse Models of Fat Embolism

One hundred twenty C57BL/6J male mice weighing 22–24 g were purchased from Shanghai SLAC Laboratory Animal Co. Ltd. and used in the present study. Ninety-six mice were randomly divided into control and fat embolism groups 1–5 (*n* = 16 per group). Injectable fat was gathered from perinephric fat from allogeneic mice and homogenized to rupture the cell membrane, then centrifuged. Mice in the fat embolism groups were intravenously injected with 2 μL/g fat. Lung tissue was obtained 4, 6, 12, 24 and 48 h after fat injection. Sixteen mice were intravenously injected with the same volume of sterile saline as the control group. Pentobarbital at a dosage of 70 mg/kg was intraperitoneally injected as anesthesia. For protein function experiments, the AQP1 inhibitor bumetanide (Sigma, St. Louis, MO, USA) was dissolved in a NaOH liquor at a concentration of 2.25 g/L and intraperitoneally injected at a dose of 0.1 mg/g before fat injection, another AQP1 inhibitor Acetazolamide (Sigma) was intragastrically administered at a volume of 0.1 mL per mice (40 mg·kg^−1^·d^−1^), according to the manufacturer’s instructions.

### 4.3. Measurement of Wet-to-Dry (W/D) Lung Weight

Lung tissue was excised from mice and weighed to obtain lung wet weights. The obtained tissues were dried in a 60 °C oven with desiccant for 72 h. Samples were then reweighed and the *W*/*D* ratio was determined.

### 4.4. Primary Cell Isolation and Culture

Pulmonary microvascular endothelial cells (PMVECs) were isolated and cultured according to previous reports [27,28]. In brief, rats were intraperitoneally injected with pentobarbital sodium and then euthanized. After thoracotomy, the lung circulatory system was perfused with an injection of 50 mL ice-cold phosphate-buffered saline (PBS) via the right ventricle. Lungs were separated and washed with 30 mL ice-cold serum-free endothelial cell medium (ScienCell Research, Carlsbad, CA, USA). Cut the pleura away, and sections (1 mm) were cut from the outer edge of the remaining lung tissue and then trimmed into small pieces. These sections were then inserted into culture bottles and rinsed in an endothelial cell medium supplemented with 20% fetal calf serum, 100 U/mL penicillin/streptomycin, and 2.5 g/mL amphotericin B. Culture bottles were incubated at 37 °C in a humidified atmosphere containing 5% CO_2_ for 60 h. Culture media were replaced every three days. Cobblestone morphology was observable and fluorescein isothiocyanate (FITC)-*Bandeiraea simplicifolia* isolectin (BSI; Sigma) were used to identify endothelial cells by immunofluorescence. The third to fifth cell passages were used for the following experiments.

### 4.5. Immunofluorescence

For immunofluorescence studies, PMVECs were arrayed on adhesive coated sections. All sections were washed twice in PBS and fixed in 4% paraformaldehyde in PBS for 30 min and permeabilized with 0.2% Triton X-100. They were then incubated overnight at 4 °C with FITC-BSI (1:100; Sigma). After labeling, sections were incubated with DAPI (Beyotime Biotech, Shanghai, China) for 3 min to stain nuclei. Images were captured by a fluorescence microscope.

### 4.6. FFA Treatment

To treat PMVECs cells with FFAs, a mixture consisting of 25% sodium-palmitic acid, 25% sodium-oleic acid, 25% arachidonic acid and 25% stearic acid (Sigma-Aldrich, Buchs, Switzerland) which dissolved in NaOH solution was added to fresh ECM at various concentrations and for various times. The cells were then harvested for RNA or protein isolation.

### 4.7. Western Blotting

Protein from cell lysates or tissues lysates were separated by SDS-polyacrylamide gel electrophoresis, transferred onto polyvinylidenedifluoride membranes, and incubated with primary antibodies, including aquaporin1 (1:800; Merck Millipore Company, Billerica, MA, USA), p38 (1:1000; Abcam, Cambridge, MA, USA), p-p38 (1:1000; Abcam), and β-actin (1:2000; Boster, China). This was followed by incubation with horseradish peroxidase (HRP)-conjugated secondary antibody (1:2000; Santa). Proteins were visualized by enhanced chemiluminescence. For kinase inhibition experiments, cells were pretreated with 10 μM SB203580 (Abcam).

### 4.8. Quantitative Real-Time PCR

Total RNA was purified from cancer cells using TRIzol (Invitrogen, Carlsbad, CA, USA) according to the manufacturer’s instructions. RNA was used for first-strand cDNA synthesis with a Takara RNA PCR Kit (Takara, Dalian, China). The resulting DNA was analyzed by quantitative real-time PCR with SYBR Premix Ex Taq (Takara) according to the manufacturer’s instructions. β-actin was used as an internal control. Primers used were as follows: AQP1 forward, 5′-GCACCTCACTCCTTTGACA-3′; AQP1 reverse, 5′-CAGAATCCCAGGCACCTAA-3′; β-actin forward, 5′-GCGTCCACCCGCGAGTACAA-3′; β-actin reverse, 5′-ACATGCCGGAGCCGTTGTCG-3′.

### 4.9. Immunohistochemistry

Paraffin-embedded sections were heated at 50 °C for 1 h, followed by microwave antigen retrieval using sodium citrate (pH = 6). They were then exposed to 1% H_2_O_2_ (prepared in methanol) at room temperature for 5 min, blocked with a sealing fluid at room temperature for 10 min, and incubated with AQP1 antibody (1:500; Merck Millipore Company) at room temperature for 30 min. After the biotinylated antibody incubation according to kit instructions, washed slices were visualized using a 3,3-diaminobenzidine-etrahydrochloride (DAB) kit (Wuhan Boster Biotechnology Corporation, Wuhan, China). This process was controlled under the microscope. Sections were counterstained with Mayer’s hematoxylin and then examined under an optical microscope. For hematoxylin and eosin stain (H & E stain), sections were stained with hematoxylin, rinsed with water, then stained with eosin, dehydrated and mounted. For Sirius red staining, sections were stained with Sirius red at 37 °C for 25 min, followed by rinsing with 100% ethanol, dehydrated and mounted.

### 4.10. Statistical Analyses

The results are expressed as means ± standard deviation (SD). With SPSS 13.0 (SPSS Institute, Chicago, IL, USA), one-way ANOVA tests for overall difference among groups, if the ANOVA turns out to be significant, then the dunnett tests for difference between the experimental groups with the control group. *p*-Values less than 0.05 were considered statistically significant.

## 5. Conclusions

Our experimental protocol has several limitations. First, we chose bumetanide (AqB013) because it is an aquaporin ligand and can block the AQP1 ion conductance [29]. However, the bumetanide derivative AqB013 also has some effect on blocking the Na-K-2Cl co-transporter [30]. Another AQP1 inhibitor, acetazolamide, functionally inhibited the osmotic water permeability of AQP1 instead of reducing AQP1 protein expression [31]. In our study, these two kinds of AQP1 inhibitors relieved pulmonary edema, although they are not specific for AQP1 inhibition. While no specific AQP1 inhibition has ever been reported, further studies are required to assess whether edema is relieved in pulmonary-specific AQP1-knockout mice. Thus, further studies are required to assess whether specific AQP1 inhibition relieves edema. Second, a study showed that after hemolysin exposure, AQP1 was no longer found on the plasma membrane, but it was found in a population of submembrane vacuoles [32]. However, there were no further experiments on the subcellular localization of AQP1 in our current study. We just demonstrated that AQP1 expression alteration was related to FFA exposure. The other limitation was that water transport in the lung involves a variety of AQPs, Na^+^-K^+^-ATPase and other ion channels, but we only focused on the effects of AQP1 in FES. Clearly, intensive experimental efforts are needed to better understand the process and mechanism of FES.

In conclusion, our study shows that the expression of AQP1 increases in the pathological process of pulmonary edema induced by FES and that the altered expression is consistent with the severity of pulmonary edema. A potential mechanism of FES is that AQP1 could be regulated by FFAs via the p38 MAPK pathway. To a certain degree, AQP1 will be a new target in the precaution and therapy for FES.
